# Development and validation of a clinical prediction model for postcontrast acute kidney injury in patients with postoperative acute kidney injury of acute Stanford type A aortic dissection

**DOI:** 10.3389/fcvm.2026.1817670

**Published:** 2026-07-10

**Authors:** Weiwei Zhao, Min Ge, YongQing Cheng, Ming Chen, Qing Zhou, Wenkui Yu

**Affiliations:** 1 Department of Cardio-Thoracic Surgery, Nanjing Drum Tower Hospital, Affiliated Hospital of Medical School, Nanjing University, Nanjing, Jiangsu Province, China; 2 Department of Intensive Care Unit, Nanjing Drum Tower Hospital, Affiliated Hospital of Medical School, Nanjing University, Nanjing, Jiangsu Province, China

**Keywords:** acute Stanford type A aortic dissection, cardiovascular surgery, clinical prediction model, contrast medium toxicity, postcontrast acute kidney injury, postoperative acute kidney injury, renal protection

## Abstract

**Objectives:**

To identify independent risk factors for postcontrast acute kidney injury (PC-AKI) in patients with postoperative AKI (PO-AKI) following acute Stanford type A aortic dissection (ATAAD), and to develop a clinically applicable prediction model.

**Methods:**

This retrospective cohort study enrolled 604 PO-AKI patients (2014–2024, Nanjing Drum Tower Hospital) who underwent ≥1 postoperative contrast-enhanced CTA. PC-AKI was diagnosed per 2018 ESUR guidelines (sCr elevation ≥26.5 μmol/L or ≥1.5 times baseline within 48–72 h, with baseline defined as the most recent pre-CTA sCr). Three variable-selection strategies (backward stepwise AIC, LASSO and XGBoost-SHAP) were used. A multivariable logistic regression model was constructed, internally validated by bootstrap resampling (1,000 repetitions), and evaluated via AUC, calibration curves, Brier score, decision curve analysis, and clinical impact curve.

**Results:**

PC-AKI incidence was 9.8% (59/604), with striking recovery-dependent stratification: 3.5% in fully recovered PO-AKI vs. 52.5% in unrecovered PO-AKI. Independent predictors included PO-AKI stage 3 (OR = 3.144, 95% CI: 1.41–7.06) and unrecovered PO-AKI before first CTA (OR = 25.212, 95% CI: 12.57–53.49). The model exhibited good discrimination (AUC=0.848, 95% CI: 0.78–0.91) and calibration (Brier=0.057). PC-AKI was independently associated with prolonged ICU stay (RR = 1.521, 95% CI: 1.19–1.97) and incomplete renal recovery at discharge (OR = 2.554, 95% CI: 1.30–4.86), but not with 30-day mortality (*P* = 0.606).

**Conclusion:**

Dynamic PO-AKI recovery and advanced AKI stage are strongly associated with PC-AKI risk in post-ATAAD patients. The internally validated model may aid individualized risk stratification before contrast procedures in this high-risk subgroup. External validation is needed before clinical deployment.

## 1 Introduction

Postcontrast acute kidney injury (PC-AKI) is a common iatrogenic complication following contrast media exposure, defined by the 2018 ESUR guidelines as an increase in serum creatinine (sCr) of ≥26.5 μmol/L or ≥1.5 times baseline within 48–72 h (1). This complication poses substantial clinical challenges, particularly in patients with ongoing postoperative acute kidney injury (PO-AKI) that has not yet resolved after surgery. Such patients have fragile renal reserve and are undergoing dynamic recovery processes, which severely complicate PC-AKI risk assessment (2, 3).

Most existing prediction models have focused on patients with chronic kidney disease (CKD) (4–7) or those undergoing coronary interventions (8–10). However, precise PC-AKI risk stratification for patients with established PO-AKI remains an unmet clinical need. Unlike CKD, which is chronic and often irreversible, AKI is an acute process that is potentially reversible, with a dynamic recovery trajectory that cannot be captured by static estimated glomerular filtration rate (eGFR). The role of AKI recovery status in PC-AKI risk has been largely overlooked, leaving clinicians uncertain about the safety of contrast-enhanced CTA in this vulnerable population.

Acute Stanford type A aortic dissection (ATAAD) is a life-threatening cardiac surgical emergency with a high postoperative AKI (PO-AKI) incidence (30%–60%) (11, 12). ATAAD patients require frequent contrast-enhanced CTA scans for preoperative diagnosis and postoperative surveillance (13), resulting in cumulative contrast exposure. This makes them well-suited for studying PC-AKI in the setting of ongoing AKI.

This study aimed to: 1) identify independent risk factors for PC-AKI in patients with PO-AKI after ATAAD; 2) develop and internally validate a prediction model for precise PC-AKI risk stratification; 3) evaluate the short-term prognostic impact of PC-AKI.

## 2 Materials and methods

### 2.1 Informed consent and ethical approval

This study adhered to the Declaration of Helsinki and was approved by the Ethics Committee of Nanjing Drum Tower Hospital (IRB name: Ethics Committee of Nanjing Drum Tower Hospital). Written informed consent was waived because the study used de–identified data from the hospital's Aortic Dissection Clinical Registry Database, posing no additional risk to patients. This study also follows the TRIPOD statement.

### 2.2 Study population and eligibility criteria

**Figure 1:**
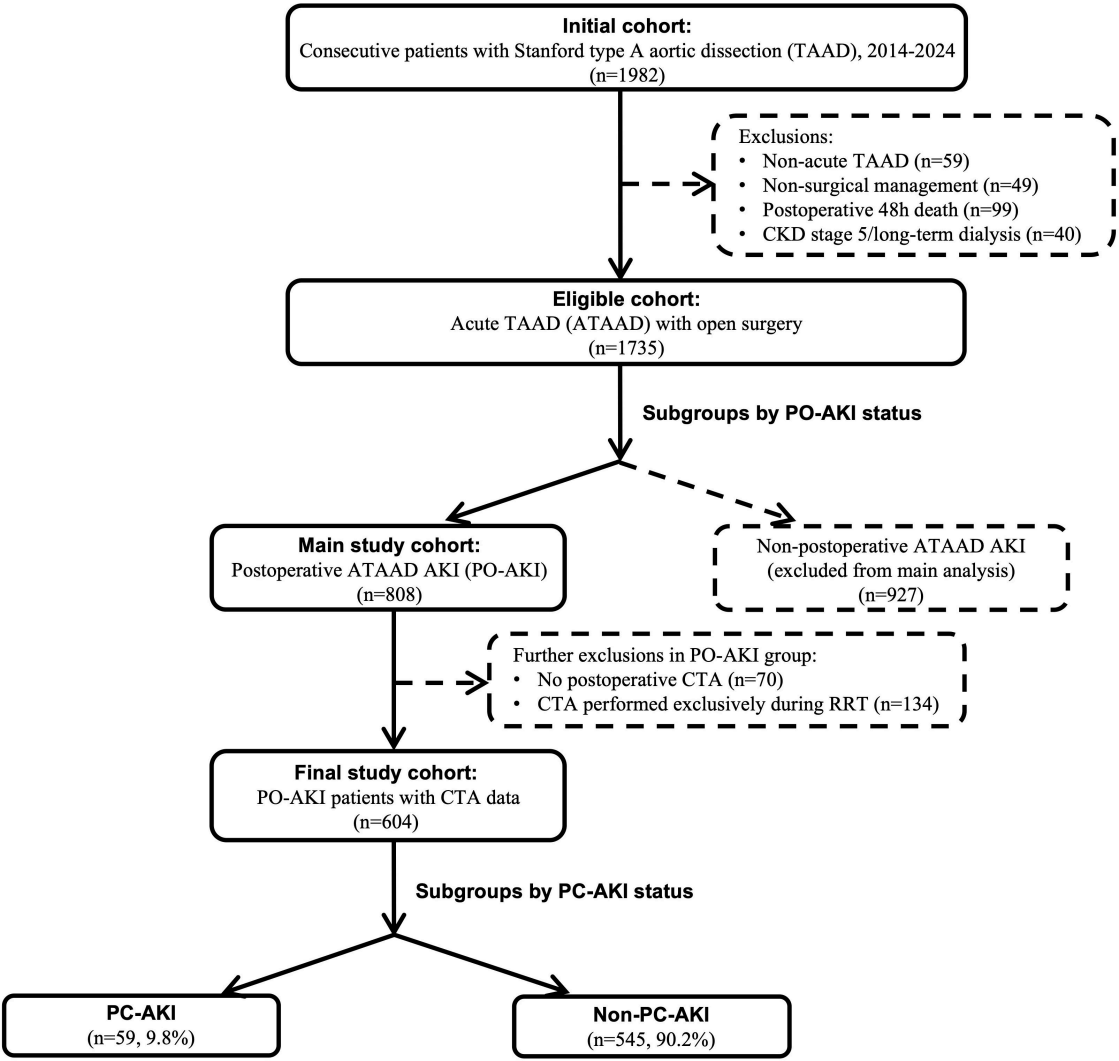
Flowchart of patient enrollment and selection. TAAD, Stanford type A aortic dissection; ATAAD, acute Stanford type A aortic dissection; AKI, acute kidney injury; CKD, chronic kidney disease; CTA, contrast-enhanced computed tomography angiography; PO-AKI, postoperative ATAAD AKI; PC-AKI, postcontrast acute kidney injury; N, number.

A total of 1,982 consecutive patients with Stanford type A aortic dissection (TAAD) (January 2014–December 2024) were initially screened (Figure 1). Exclusions included non-acute Stanford type A aortic dissection (non-ATAAD) (*n* = 59), non-surgical management (*n* = 49), 48-hour postoperative mortality (*n* = 99), and preoperative CKD stage 5/long-term dialysis (*n* = 40); Patients with CKD stages 2–4 were retained, as excluding them would introduce selection bias given the low awareness rate of mild CKD in China [AEA RCT Registry, AEARCTR-0013721, 2025] and the emergency setting. Baseline eGFR was adjusted for in all models. The remaining 1,735 surgical patients with ATAAD were stratified into postoperative AKI (PO-AKI, *n* = 808) and non-PO-AKI (*n* = 927, excluded from main analysis), with PO-AKI defined per KDIGO criteria (14). Further exclusions from the PO-AKI group included patients with no postoperative CTA (*n* = 70, early death, transfer, or discharge) and those underwent CTA during renal replacement therapy (RRT) (*n* = 134), because RRT alters contrast metabolism and sCr clearance, which may confound PC-AKI diagnosis (1).

### 2.3 Study definitions and diagnostic criteria

Acute Stanford type A aortic dissection (ATAAD) was defined as a dissection involving the ascending aorta with symptom onset within 14 days (15). In the setting of emergency ATAAD surgery, outpatient prior serum creatinine (sCr) records were not available for all patients. Baseline renal function was therefore defined using the admission sCr (or eGFR) obtained immediately before surgery. Chronic kidney disease (CKD) was defined as structural renal damage or eGFR <60 mL/(min·1.73 m²) for ≥3 months (16). Postoperative acute kidney injury (PO-AKI) was defined as any of the following: sCr elevation ≥26.5 μmol/L within 48 h, ≥1.5–fold increase from baseline within 7 days, urine output <0.5 mL/(kg·h) for 6 h, or initiation of renal replacement therapy (RRT) postoperatively (14, 17). PO–AKI recovery status before CTA was assessed based on the patient's sCr trajectory relative to the admission baseline, the peak postoperative sCr value, and the dynamic change, and was classified as: Fully recovered: sCr ≤ admission baseline + 0.3 mg/dL; Partially recovered: sCr > admission baseline + 0.3 mg/dL but decreased by ≥25% from the peak postoperative sCr value; Unrecovered: sCr decreased by <25% or unimproved from the peak value, or still requiring RRT (18–20).

Postcontrast acute kidney injury (PC-AKI) was defined per the 2018 ESUR guidelines as sCr elevation ≥26.5 μmol/L or ≥1.5–fold relative to the most recent pre–CTA sCr measurement, occurring within 48–72 h after contrast exposure (1).

### 2.4 Data collection and study outcomes

Demographic data (age, sex, BMI, smoking, drinking), medical history (hypertension, diabetes, CAD, stroke, CKD), admission parameters (sCr, limb hypoperfusion), surgical data (duration, CPB time, aortic cross-clamp time, Deep Hypothermic Circulatory Arrest time, RBC transfusion), CTA-related features (frequency, timing relative to surgery and PO-AKI onset, type, and interval between consecutive scans), AKI staging/recovery status, and short-term outcomes (renal recovery, ICU/hospital stay, 30-day all-cause mortality) were extracted. The primary outcome was PC-AKI, and the secondary outcomes included: ICU stay, hospital stay, 30-day all-cause mortality, and renal recovery at discharge (fully/partially/unrecovered, as defined in Section 2.3).

### 2.5 Statistical analysis

#### 2.5.1 Sample size calculation for prediction model development

Sample size requirements for prediction model development were assessed according to the framework proposed by Riley et al. (21) using the pmsampsize package in R. For a binary outcome, the calculation incorporated an anticipated Nagelkerke's R^2^ of 0.50, 15 candidate predictor parameters, an expected PC-AKI prevalence of 10%, and a desired shrinkage factor of 0.90. Based on these assumptions, the minimum required sample size was 541 participants, including at least 55 outcome events.

#### 2.5.2 Descriptive statistical analysis

The distribution of continuous variables was assessed using the Shapiro–Wilk test for normality. Normally distributed variables were presented as mean ± standard deviation and compared using the independent samples t-test; non-normally distributed variables were presented as median [interquartile range] and compared using the Mann–Whitney U test. Categorical variables were presented as frequency (percentage) and compared using the chi-square test.

#### 2.5.3 Development and validation

The primary analysis treated PC–AKI as a binary patient–level outcome (presence vs. absence after the first CTA). Due to the lack of an external validation cohort, the dataset was divided into training and validation sets (at 6:4 ratio) using two splitting strategies: temporal split (by admission date) and random split. This allowed us to evaluate model stability across different data partitions. In the training set, variable selection was performed using backward stepwise regression (based on the Akaike Information Criterion), LASSO regression with 10-fold cross-validation (*λ* selected as *λ*_min corresponding to the minimum cross-validated deviance), and XGBoost (based on SHAP value ranking with cumulative contribution ≥80%). Variables selected by at least two methods were identified as key predictors (Supplementary Table S1).

Model discrimination was evaluated using the area under the receiver operating characteristic curve (AUC) with 95% confidence intervals (CI), and differences in AUC between training and validation sets were assessed using DeLong's test. Calibration was assessed using calibration plots and the Brier score. Considering that data splitting may reduce statistical efficiency, the final model was refitted using the entire dataset to obtain stable regression coefficients and the final prediction formula, followed by internal validation using 1,000 bootstrap resamples. Clinical utility was evaluated using decision curve analysis (DCA) and clinical impact curves (CIC), and a nomogram was constructed for individualized risk prediction. A random seed of 123 was used for all stochastic procedures, including dataset splitting, bootstrap resampling, and model training.

#### 2.5.4 Association between PC-AKI and postoperative outcomes

To further evaluate the association between PC-AKI and postoperative outcomes, 30-day mortality, renal function recovery at discharge (recoded as 0 = full recovery, 1 = partial or no recovery), ICU length of stay, and total hospital length of stay were included as primary outcomes. Multivariable logistic regression was used for 30-day mortality and renal function recovery at discharge, with odds ratios (ORs) and 95% CIs estimated. For ICU and hospital length of stay, the Shapiro–Wilk test was first used to assess distributional characteristics. If variables approximated a normal distribution, multivariable linear regression was applied; if variables showed marked skewness, Gamma regression with a log link function was used to estimate relative effects (RRs) and 95% CIs. All models were adjusted for age, sex, BMI, smoking, drinking, hypertension, diabetes mellitus, coronary artery disease, stroke, chronic kidney disease, limb hypoperfusion, log-transformed admission serum creatinine, and postoperative CTA frequency.

In this study, only BMI and admission serum creatinine had missing values, with missing rates of 6.29% and 0.99%, respectively. Missing data were handled using multiple imputation by chained equations. All statistical analyses were performed using SPSS 26.0 and R 4.5.1, and the R packages used included mice, rms, glmnet, xgboost, shapviz, rmda, and pROC. All tests were two-sided, and *P* < 0.05 was considered statistically significant.

## 3 Results

### 3.1 Baseline characteristics and PC-AKI incidence

A total of 604 patients with PO–AKI were included after the exclusion steps detailed in Figure 1 and Methods 2.2. Baseline characteristics are summarized in Table 1. Mean age was 54.15 ± 12.96 years, 485 (80.3%) were male, and mean BMI was 26.95 ± 4.18 kg/m^2^. Overall, 59 developed PC-AKI after the first CTA (9.8%, 59/604), while the PC-AKI incidence was 3.5% in fully recovered PO-AKI but reached 52.5% in unrecovered PO-AKI. Compared with Non-PC–AKI group, those with PC–AKI were older, more often female, had a higher prevalence of preoperative stroke, more advanced PO–AKI stages, and underwent first CTA earlier (all *P* < 0.05).

**Table 1:** Baseline characteristics of patients stratified by PC-AKI status.

Variables	Total (*n* = 604)	Non-PC-AKI (*n* = 545)	PC-AKI (*n* = 59)	*P* value
Demographic Data				
Age, years	54.15 ± 12.96	53.79 ± 12.93	57.49 ± 12.83	**0**.**037**
Sex, male	485 (80.3)	445 (81.7)	40 (67.8)	**0**.**011**
BMI, kg/m^2^	26.95 ± 4.18	27.02 ± 4.21	26.30 ± 3.95	0.214
Smoking, yes	173 (28.6)	155 (28.4)	18 (30.5)	0.855
Drinking, yes	103 (17.1)	93 (17.1)	10 (16.9)	0.998
Medical History				
Hypertension, yes	480 (79.5)	428 (78.5)	52 (88.1)	0.118
Diabetes mellitus, yes	18 (3.0)	17 (3.1)	1 (1.7)	0.835
Coronary artery disease, yes	28 (4.6)	26 (4.8)	2 (3.4)	0.878
Stroke, *n* (%)	23 (3.8)	17 (3.1)	6 (10.2)	**0**.**007**
Chronic kidney disease, yes	20 (3.3)	17 (3.1)	3 (5.1)	0.676
Admission sCr (*μ*mol/L)	84 [66, 105]	84 [66, 105]	84 [65.7, 105.1]	0.969
Admission limb hypoperfusion, yes	12 (2.0)	9 (1.7)	3 (5.1)	0.192
Surgical Data				
Surgery duration, min	453.54 ± 106.71	456.44 ± 107.13	426.80 ± 99.68	0.043
Cardiopulmonary bypass (CPB) time, min	221.53 ± 61.64	222.14 ± 61.23	215.86 ± 65.55	0.458
Aortic cross-clamp time, min	156.93 ± 49.69	156.70 ± 49.28	159.05 ± 53.73	0.730
Deep hypothermic circulatory arrest (DHCA) time, min	28.80 ± 10.27	28.87 ± 10.20	28.16 ± 10.99	0.623
Intraoperative RBC transfusion volume, mL	2,000 [1,500, 2,800]	2,000 [1,500, 2,700]	2,000 [1,575, 2,910]	0.302
PO-AKI Related Characteristics				
PO-AKI stage, *n* (%)				**<0**.**001**
Stage 1	315 (52.2)	295 (54.1)	20 (33.9)	
Stage 2	155 (25.7)	141 (25.9)	14 (23.7)	
Stage 3	134 (22.2)	109 (20.0)	25 (42.4)	
PO-AKI recovery status before 1st CTA, *n* (%)				**<0**.**001**
Fully recovered	374 (61.9)	361 (66.2)	13 (22.0)	
Partially recovered	150 (24.8)	146 (26.8)	4 (6.8)	
Unrecovered	80 (13.2)	38 (7.0)	42 (71.2)	
Time interval from surgery and 1st CTA, days	7 (3, 11)	7 (4, 11)	1 (0, 7)	**<0**.**001**
Postoperative CTA frequency, *n* (%)				**<0**.**001**
1 scan	503 (83.3)	483 (88.6)	20 (33.9)	
≥2 scans	101 (16.7)	62 (11.4)	39 (66.1)	
Short-Term Outcomes				
30-day mortality, *n* (%)	23 (3.8)	21 (3.9)	2 (3.4)	0.998
Renal function recovery at discharge, *n* (%)				**<0**.**001**
Fully recovered	511 (84.6)	468 (85.9)	43 (72.9)	
Partially recovered	84 (13.9)	72 (13.2)	12 (20.3)	
Unrecovered	9 (1.5)	5 (0.9)	4 (6.8)	
ICU stays, days	6 [4, 9]	5 [4, 8]	9 [6, 15]	**<0**.**001**
Hospital stays, days	19 [16, 25]	19 [16, 25]	20 [16.5, 32]	0.070

Bold indicate *P* < 0.05. Continuous variables were expressed as mean ± SD (normal distribution) or median [IQR] (non-normal distribution), compared via T-test or Mann–Whitney U test. Categorical variables were expressed as *n* (%), compared via *χ²* or Fisher's exact test. PC-AKI, postcontrast acute kidney injury; PO-AKI, postoperative AKI; sCr, serum creatinine; RBC, red blood cell; RRT, renal replacement therapy; ICU, intensive care unit.

In addition, 102 patients underwent a second CTA, 14 a third, and 2 a fourth. Of the 14 patients who underwent ≥3 CTAs, 11 were fully recovered at the time of their repeat scans. Five patients experienced recurrent PC-AKI episodes after their second CTA examinations. Among the 31 patients who received RRT, 29 had a postoperative CTA after RRT discontinuation, and two of these 29 (6.9%) developed PC–AKI. Both had unrecovered AKI and underwent CTA within 6 days after RRT withdrawal.

### 3.2 Development and performance of the PC-AKI prediction model

#### 3.2.1 Variable selection

Supplementary Figures S1,S2 present the results of variable selection using LASSO regression and XGBoost, respectively. Supplementary Table S1 summarizes the predictors selected by three methods under both temporal-split and random-split validation strategies. Among all candidate predictors, PO-AKI recovery status before the first CTA was selected six times, PO-AKI stage five times, and BMI three times. All other variables were selected no more than twice and only under specific data-splitting strategies or variable-selection methods. PO-AKI recovery status, PO-AKI stage, and BMI were initially included in a multivariable logistic regression model. However, BMI was not independently predictive in multivariable analysis (β = 0.010, *P* = 0.806). Therefore, BMI was excluded from the final model, leaving only PO-AKI recovery status and PO-AKI stage as key predictors.

#### 3.2.2 Model performance and validation

Under temporal-split validation, the model achieved an AUC of 0.890 (95% CI: 0.80–0.98) in the training set and 0.825 (95% CI: 0.74–0.91) in the validation set, with no significant difference between cohorts (D = 1.019, *P* = 0.309). Random-split validation yielded similar results: training AUC 0.882 (95% CI: 0.79–0.97) and validation AUC 0.817 (95% CI: 0.73–0.91) (D = 0.994, *P* = 0.320).

**Figure 2:**
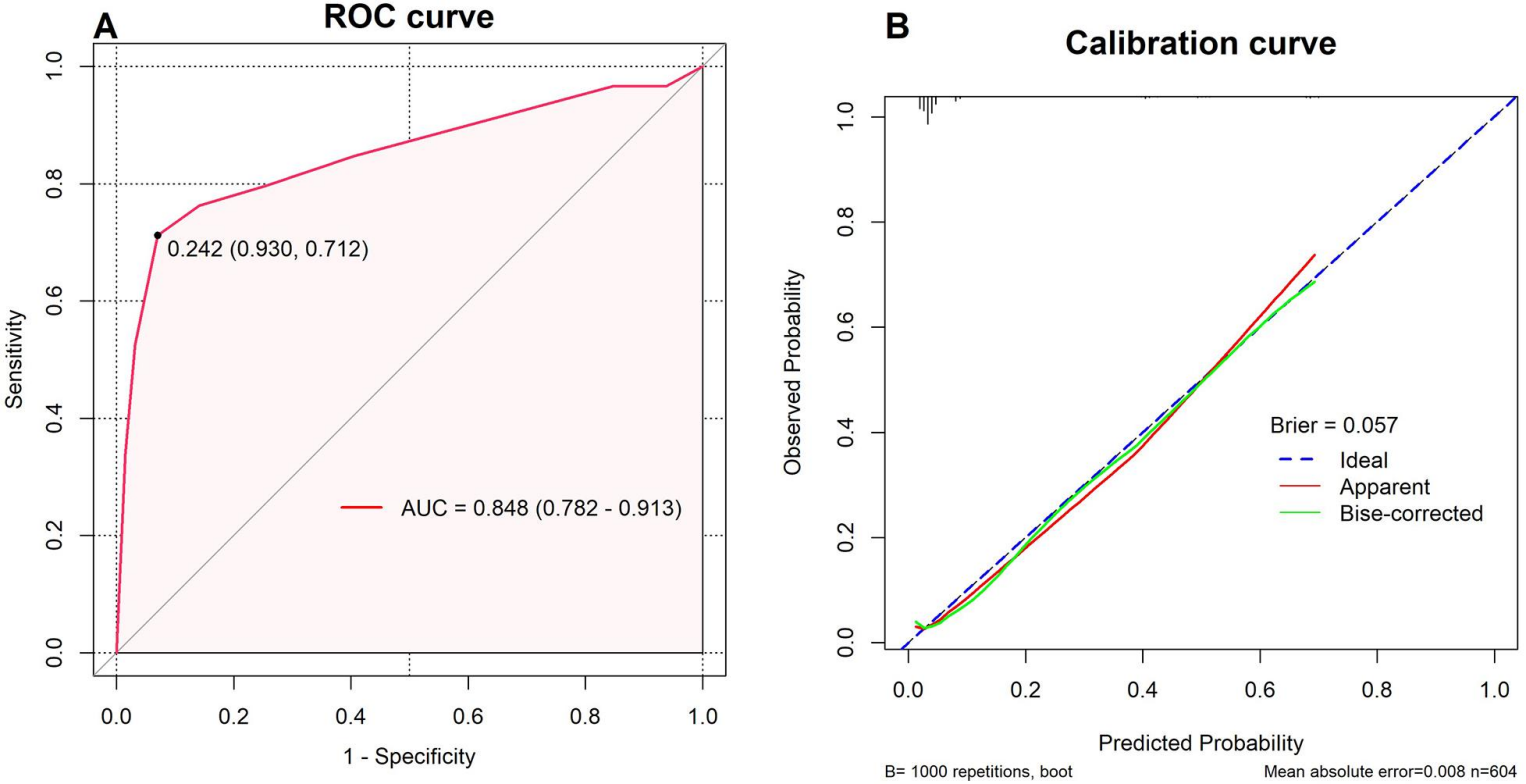
Discrimination and calibration performance of the final PC-AKI prediction model. **(A)** ROC curve; **(B)** calibration curve.

To preserve statistical power, the final model was refitted on the entire dataset. As shown in Figure 2A, the final model demonstrated good discrimination (AUC=0.848, 95% CI: 0.78–0.91). Internal validation using 1,000 bootstrap resamples gave an optimism-corrected AUC of 0.836 (95% CI: 0.78–0.91). The calibration curve (Figure 2B) showed good agreement between predicted and observed risks. Bootstrap validation yielded a calibration slope (shrinkage factor) of 0.965 and an intercept of −0.046, indicating minimal overfitting. The apparent Brier score was 0.057, and the bootstrap-corrected Brier score was 0.057 (95% CI: 0.05–0.07), supporting model's stability.

The final prediction model is: Linearpredictor=−3.607+ 0.357×Stage2+1.145×Stage3−0.706×PartialRecovery+ 3.227×Unrecovered(where Stage2/Stage3 indicate PO–AKI stage 2 and 3 (reference: stage 1), and Partial Recovery/Unrecovered indicate partial or no recovery before first CTA (reference: fully recovered). Predicted $\mathrm{PCAKI}\;\mathrm{probability}=\frac{\exp(\mathrm{Linear}\;\mathrm{predictor})}{1+\exp(\mathrm{Linear}\;\mathrm{predictor})}$. Multivariable logistic regression results are presented in Table 2.

**Table 2:** Association of key predictors with PC-AKI.

Predictors	*β*	P	OR	95% CI
Intercept	−3.607	<0.001	-	-
PO-AKI Stage				
Stage 1	Ref.	-	-	-
Stage 2	0.357	0.403	1.429	0.61–3.28
Stage 3	1.145	0.005	3.144	1.41–7.06
PO-AKI recovery status before 1st CTA				
Fully recovered	Ref.	-	-	-
Partially recovered	−0.706	0.242	0.493	0.13–1.50
Unrecovered	3.227	<0.001	25.212	12.57–53.49

OR, odds ratio; CI, confidence interval; Ref, reference group.

#### 3.2.3 Clinical utility of the PC-AKI prediction model

**Figure 3:**
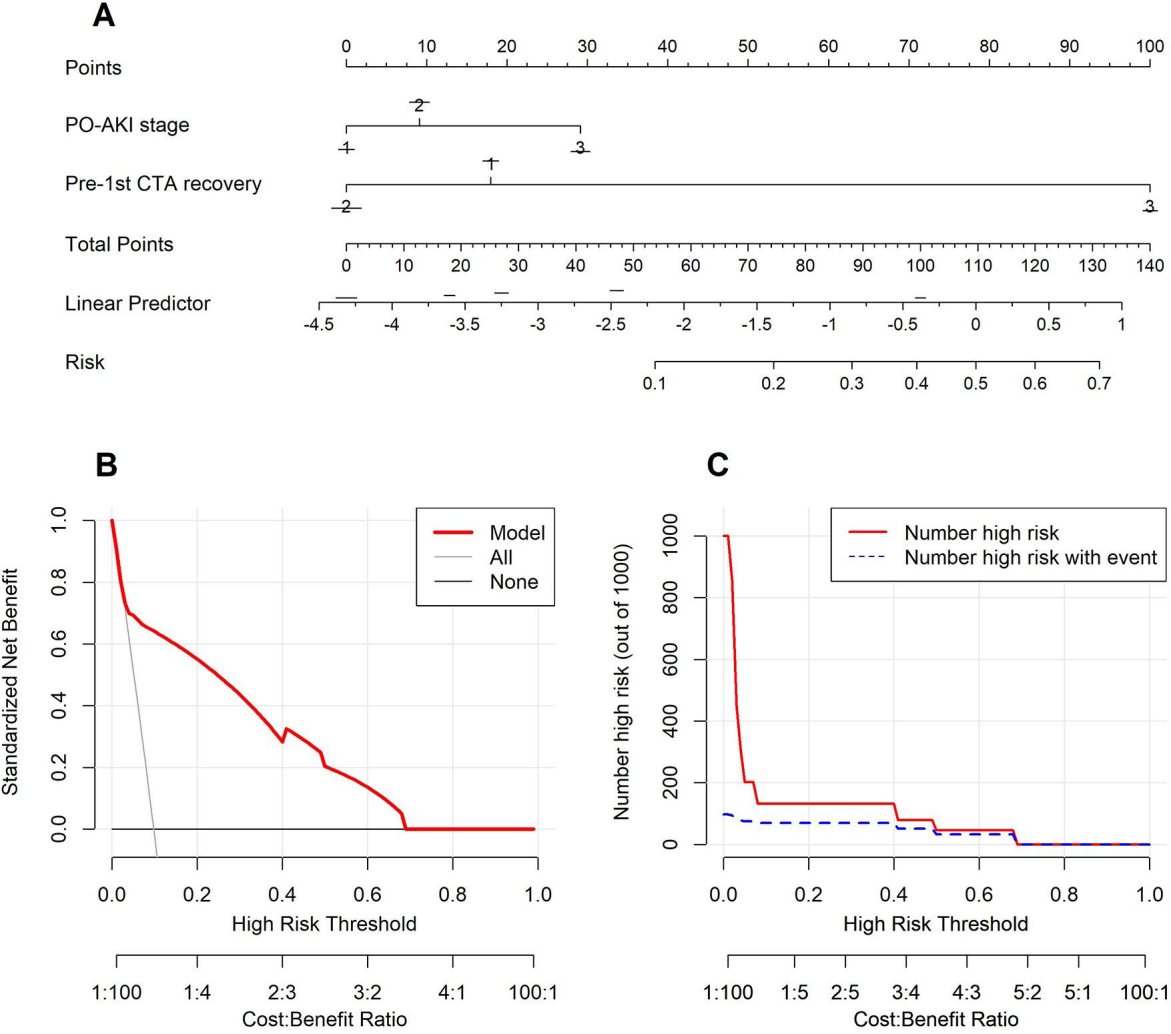
Nomogram **(A)**, decision curve **(B)** and clinical impact curve **(C)** for the PC-AKI prediction model.

The final prediction model was presented as a nomogram (Figure 3A). The point assignment for each predictor is in Supplementary Table S6. Figures 3B,C show the decision curve analysis (DCA) and clinical impact curve (CIC), respectively. At risk thresholds of 0.2, 0.3, and 0.4, the net benefits were 0.054, 0.043, and 0.028, corresponding to 54, 43, and 28 additional correct interventions per 1,000 patients, with 7% of patients classified as high-risk at each threshold. Compared with the “treat all” strategy (net benefits −0.128 to −0.504), the model reduced unnecessary interventions.

### 3.3 Association between PC-AKI and postoperative outcomes

Table 3 summarizes the associations between PC-AKI and postoperative outcomes. Patients with PC-AKI were not at significantly increased risk of 30-day mortality. However, PC-AKI was significantly associated with impaired renal function recovery at discharge (OR = 2.554, 95% CI: 1.30–4.86). The distributions of ICU and hospital stay were markedly right-skewed (skewness 2.98 and 2.56, respectively); therefore, gamma regression with a log link was applied. PC-AKI was associated with prolonged ICU stay (RR = 1.521, 95% CI: 1.19–1.97) and longer hospital stay (RR = 1.151, 95% CI: 1.01–1.32). Complete results are provided in Supplementary Tables S2–S5.

**Table 3:** Associations of PC-AKI with postoperative outcomes.

Outcomes	β	P	OR/RR^a^	95% CI
30-day mortality^b^	−0.404	0.606	0.667	0.10–2.52
ICU stay	0.419	<0.001	1.521	1.19–1.97
Renal function recovery at discharge	0.938	0.005	2.554	1.30–4.86
Hospital stay	0.140	0.039	1.151	1.01–1.32
30-day mortality^b^	−0.404	0.606	0.667	0.10–2.52

a 30-day mortality and renal function recovery at discharge were analyzed using logistic regression; ICU stay and hospital stay, which were right-skewed, were analyzed using Gamma regression with a log link.

b 30-day mortality model excluded diabetes mellitus and CKD due to zero events in these subgroups. Models were adjusted for age, sex, BMI, smoking, drinking, hypertension, diabetes mellitus, coronary artery disease, stroke, chronic kidney disease, admission limb hypoperfusion, and log-transformed admission serum creatinine.

With 3 deaths in the PC–AKI group (*n* = 59) and 20 in the non–PC–AKI group (*n* = 541), the study had 80% power to detect an odds ratio ≥4.5 (*α*=0.05). Thus, the lack of a significant association (OR 0.667, 95% CI 0.10–2.52) does not exclude smaller but clinically meaningful mortality differences.

## 4 Discussion

This is the first study to develop a PC–AKI prediction model specifically for patients with PO–AKI after ATAAD. The model demonstrated good discrimination (optimism–corrected AUC 0.836) and calibration (Brier 0.057), addressing an unmet clinical need in a population where existing tools — largely derived from CKD or coronary intervention cohorts — are not directly applicable.

The 9.8% PC–AKI incidence in our cohort reflects an intermediate risk spectrum, driven by ongoing AKI and cumulative low–volume contrast exposure. More importantly, unrecovered PO–AKI before first CTA emerged as the strongest predictor (OR = 25.21), far exceeding PO–AKI stage 3 (OR = 3.14). This disparity underscores that the dynamic trajectory of renal recovery — rather than the static severity of AKI — is the dominant determinant of PC–AKI risk. This pattern is further reflected in the observation that, among the 29 patients who underwent CTA after RRT discontinuation, only 2 (6.9%) developed PC–AKI — though this finding is descriptive and derived from a small sample. Consistent with the concept of a “vulnerability window” during AKI recovery, marked by persistent renal inflammation and hypoperfusion (22), our results suggest that renal recovery attenuates susceptibility to subsequent contrast insult. A recent study applying machine learning to predict PC–AKI in CKD patients also highlighted the importance of dynamic renal function changes (8), supporting our emphasis on recovery status over static eGFR.

The recovery–dependent risk gradient in our cohort is clinically striking: fully recovered patients had a PC–AKI risk (3.5%) approximating that of the general population (0.6%–2.3%) (23), whereas unrecovered patients showed a risk (52.5%) exceeding that reported in CKD, diabetic, and STEMI cohorts (4, 24–26). This wide spectrum within a single ATAAD–PO–AKI population demonstrates that PO–AKI recovery status is a powerful clinical discriminator — capable of stratifying patients from near–baseline risk to extreme risk. Recent studies have proposed composite indices (e.g., nutritional–inflammatory scores, hemodynamic burden indices) for PC–AKI risk stratification in other populations (27, 28), but our findings suggest that in the post–ATAAD setting, the recovery trajectory itself outperforms any static composite measure. This provides a practical basis for individualized decision–making: patients with unrecovered PO–AKI — particularly stage 3 — should be prioritized for risk mitigation before any contrast procedure.

Several methodological considerations warrant attention. First, distinguishing a new PC–AKI episode from an ongoing PO–AKI trajectory is inherently challenging in clinical practice, as both rely on serum creatinine changes. We addressed this by defining the PC–AKI baseline as the most recent pre–CTA sCr, so that diagnosis requires a rise from the immediate pre-exposure value rather than from the original PO–AKI baseline. This follows the 2018 ESUR guideline. Second, the earlier CTA timing in PC–AKI patients (median 1 vs. 7 days) suggests confounding by indication: sicker patients were scanned earlier. The 2018 ESUR nomenclature shift from CIN to PC–AKI explicitly acknowledges that post–contrast renal deterioration cannot be unequivocally attributed to contrast medium itself, as multiple factors may contribute. While this limitation is inherent to all creatinine–based PC–AKI studies, we are conducting prospective studies incorporating novel biomarkers to better differentiate mechanisms of sequential renal injury.

This study has several limitations. First, its retrospective single–center design and single–ethnicity (Chinese) cohort may limit generalizability and introduce selection bias. We lacked data on fluid balance, per–scan contrast volume, nephrotoxic medications, serum electrolyte levels, and urinary biomarkers (NGAL, KIM-1, cystatin C), which could lead to residual confounding (29, 30). Second, the absence of long-term follow-up limits evaluation of CKD progression. Future multicenter prospective studies should validate our model, integrate novel AKI biomarkers, and examine whether model-guided CTA decisions reduce long-term renal dysfunction.

We propose a risk–adapted framework for PC–AKI prevention: prioritize unrecovered PO–AKI (notably stage 3) for risk stratification, and avoid unnecessary contrast procedures in this subgroup. Decisions to delay repeat CTA should be individualized based on predicted PC–AKI risk and clinical urgency, as no evidence–based fixed interval could be derived from our data. This precision–guided strategy aligns diagnostic needs with renal protection.

## Data Availability

The original contributions presented in the study are included in the article/Supplementary Material, further inquiries can be directed to the corresponding authors.

## Supplementary material

The Supplementary Materialfor this article can be found online at: https://www.frontiersin.org/articles/10.3389/fcvm.2026.1817670/full#supplementary-material

Supplementary Table S1 Variables selected by different feature selection methods under temporal-split and random-split validation strategies.

Supplementary Table S2 Association between PC-AKI and 30-day mortality.

Supplementary Table S3 Association between PC-AKI and renal function recovery at discharge.

Supplementary Table S4 Association between PC-AKI and ICU stay.

Supplementary Table S5 Association between PC-AKI and hospital stay.

Supplementary Table S6 Point assignment based on the nomogram for PC-AKI prediction.

Supplementary Figure S1 LASSO regression for predictor selection in PC-AKI models.

Supplementary Figure S2 XGBoost feature selection for PC-AKI prediction using SHAP values.
