# Supplementary material for: Development and validation of a clinical prediction model for postcontrast acute kidney injury in patients with postoperative acute kidney injury of acute Stanford type A aortic dissection
Source: Front Cardiovasc Med. 2026 Jul 10;13:1817670. doi: 10.3389/fcvm.2026.1817670 (PMC13397107; doi:10.3389/fcvm.2026.1817670)
Supplement: Supplementary Table S1 — Variables selected by different feature selection methods under temporal-split and random-split validation strategies. [file Supplementaryfile1.docx]

**Supplementary materials**

**Development and Validation of a Clinical Prediction Model for Postcontrast Acute Kidney Injury in Patients with Postoperative-Acute Kidney Injury of Acute Stanford Type A Aortic Dissection**

Weiwei Zhao^1†^, Min Ge^1†^, YongQing Cheng^1†^, Ming Chen^2, *^, Qing Zhou^1, *^, Wenkui Yu^2, *^

1 Department of Cardio-Thoracic Surgery, Nanjing Drum Tower Hospital, Affiliated Hospital of Medical School, Nanjing University, Nanjing, 210008, Jiangsu Province, China.

2 Department of Intensive Care Unit, Nanjing Drum Tower Hospital, Affiliated Hospital of Medical School, Nanjing University, Nanjing, 210008, Jiangsu Province, China.

† These authors contributed equally to this work and share the first authorship.

* These authors contributed equally to this work and share the corresponding authorship.

**Correspondence to**: Dr Wenkui Yu, PhD, MD, Department of Intensive Care Unit, Nanjing Drum Tower Hospital, Affiliated Hospital of Medical School, Nanjing University, Nanjing, 210008, Jiangsu Province, China (Email: [yudrnj2@163.com](mailto:yudrnj2@163.com)).

**Contents**

**Supplementary Tables**

Table S1. Variables selected by different feature selection methods under temporal-split and random-split validation strategies

Table S2. Association between PC-AKI and 30-day mortality

Table S3. Association between PC-AKI and renal function recovery at discharge

Table S4. Association between PC-AKI and ICU stay

Table S5. Association between PC-AKI and hospital stay

Table S6. Point assignment based on the nomogram for PC-AKI prediction

**Supplementary Figures**

Figure S1. LASSO regression for predictor selection in PC-AKI models.

Figure S2. XGBoost feature selection for PC-AKI prediction using SHAP values.

**Table S1.** Variables selected by different feature selection methods under temporal-split and random-split validation strategies

| Candidate variable | AIC | |  | LASSO | |  | XGBoost | | Count |
| --- | --- | --- | --- | --- | --- | --- | --- | --- | --- |
|  | Temp | Rand |  | Temp | Rand |  | Temp | Rand |  |
| PO‑AKI recovery status | ✓ | ✓ |  | ✓ | ✓ |  | ✓ | ✓ | 6 |
| PO‑AKI stage | ✓ |  |  | ✓ | ✓ |  | ✓ | ✓ | 5 |
| BMI |  | ✓ |  |  |  |  | ✓ | ✓ | 3 |
| Drinking | ✓ |  |  | ✓ |  |  |  |  | 2 |
| Age |  |  |  |  |  |  | ✓ | ✓ | 2 |
| Time interval: surgery to 1st CTA |  |  |  |  |  |  | ✓ | ✓ | 2 |
| Hypertension |  | ✓ |  |  | ✓ |  |  |  | 2 |
| Stroke |  |  |  |  | ✓ |  |  |  | 1 |
| CAD |  | ✓ |  |  |  |  |  |  | 1 |
| Admission log(sCr) |  |  |  |  |  |  | ✓ |  | 1 |

**Note:** Temp = temporal split; Rand = random split. AIC = backward stepwise regression based on Akaike Information Criterion; LASSO = least absolute shrinkage and selection operator with 10‑fold cross‑validation; XGBoost = extreme gradient boosting with SHAP value ranking (cumulative contribution ≥80%). "Count" indicates the number of method‑split combinations in which the variable was selected. Variables with Count ≥2 were considered for the final model. Among these, PO‑AKI recovery status, PO‑AKI stage, and BMI were initially included in multivariable logistic regression; BMI was not independently predictive (β = 0.010, P = 0.806) and was therefore excluded from the final model.

**Table S2.** Association between PC-AKI and 30-day mortality

| Independent variable | *β* | *Se* | *P* |
| --- | --- | --- | --- |
| Intercept | -6.684 | 3.483 | 0.055 |
| Age | **0.047** | **0.020** | **0.018** |
| Gender (female) | -0.369 | 0.575 | 0.521 |
| BMI | 0.014 | 0.059 | 0.808 |
| Smoking (yes) | 0.218 | 0.613 | 0.722 |
| Drinking (yes) | -0.343 | 0.772 | 0.657 |
| Hypertension (yes) | 0.535 | 0.642 | 0.405 |
| CAD (yes) | 0.401 | 0.799 | 0.616 |
| Stroke (yes) | 0.534 | 0.823 | 0.516 |
| Admission limb hypoperfusion | 1.180 | 1.107 | 0.287 |
| Log-transformed admission sCr | 0.067 | 0.590 | 0.910 |
| PC-AKI (yes) | -0.404 | 0.783 | 0.606 |

Note: *Se*, standard error. This model excluded diabetes mellitus and CKD due to zero events in these subgroups. Bold indicates *P* < 0.05.

**Table S3**. Association between PC-AKI and renal function recovery at discharge

| Independent variable | *β* | *Se* | *P* |
| --- | --- | --- | --- |
| Intercept | 3.329 | 2.015 | 0.098 |
| Age | -0.007 | 0.011 | 0.508 |
| Gender (female) | -0.383 | 0.352 | 0.277 |
| BMI | 0.001 | 0.031 | 0.976 |
| Smoking (yes) | 0.445 | 0.304 | 0.143 |
| Drinking (yes) | -0.215 | 0.359 | 0.549 |
| Hypertension (yes) | -0.034 | 0.291 | 0.905 |
| Diabetes (yes) | 0.401 | 0.623 | 0.520 |
| CAD (yes) | 0.788 | 0.474 | 0.097 |
| Stroke (yes) | -0.751 | 0.784 | 0.337 |
| CKD (yes) | 0.784 | 0.614 | 0.202 |
| Admission limb hypoperfusion | 0.544 | 0.707 | 0.441 |
| Log-transformed admission sCr | **-1.017** | **0.364** | **0.005** |
| PC-AKI (yes) | **0.938** | **0.335** | **0.005** |

Note: *Se*, standard error. Bold indicates *P* < 0.05.

**Table S4**. Association between PC-AKI and ICU stay

| Independent variable | *β* | *Se* | *P* |
| --- | --- | --- | --- |
| Intercept | -0.127 | 0.620 | 0.837 |
| Age | **0.007** | **0.003** | **0.021** |
| Gender (female) | **0.213** | **0.105** | **0.042** |
| BMI | 0.014 | 0.009 | 0.136 |
| Smoking (yes) | 0.081 | 0.104 | 0.435 |
| Drinking (yes) | -0.016 | 0.122 | 0.890 |
| Hypertension (yes) | 0.062 | 0.092 | 0.501 |
| Diabetes (yes) | -0.117 | 0.224 | 0.600 |
| CAD (yes) | -0.110 | 0.178 | 0.535 |
| Stroke (yes) | 0.121 | 0.197 | 0.538 |
| CKD (yes) | -0.298 | 0.210 | 0.156 |
| Admission limb hypoperfusion | 0.239 | 0.268 | 0.371 |
| Log-transformed admission sCr | **0.228** | **0.106** | **0.032** |
| PC-AKI (yes) | **0.419** | **0.125** | **<0.001** |

Note: *Se*, standard error. Bold indicates *P* < 0.05.

**Table S5**. Association between PC-AKI and hospital stay

| Independent variable | *β* | *Se* | *P* |
| --- | --- | --- | --- |
| Intercept | **2.425** | **0.334** | **<0.001** |
| Age | 0.002 | 0.002 | 0.295 |
| Gender (female) | 0.009 | 0.057 | 0.873 |
| BMI | -0.004 | 0.005 | 0.441 |
| Smoking (yes) | 0.034 | 0.056 | 0.551 |
| Drinking (yes) | -0.080 | 0.066 | 0.229 |
| Hypertension (yes) | 0.021 | 0.050 | 0.672 |
| Diabetes (yes) | 0.086 | 0.121 | 0.476 |
| CAD (yes) | -0.167 | 0.096 | 0.084 |
| Stroke (yes) | -0.078 | 0.106 | 0.461 |
| CKD (yes) | -0.031 | 0.114 | 0.785 |
| Admission limb hypoperfusion | 0.034 | 0.145 | 0.815 |
| Log-transformed admission sCr | **0.145** | **0.058** | **0.012** |
| PC-AKI (yes) | **0.141** | **0.068** | **0.039** |

Note: *Se*, standard error. Bold indicates *P* < 0.05.

**Table S6.** Point assignment based on the nomogram for PC-AKI prediction

| Predictor | Points |
| --- | --- |
| PO-AKI stage 1 | 0 |
| PO-AKI stage 2 | 9 |
| PO-AKI stage 3 | 29 |
| Pre-1st CTA recovery: fully recovered | 18 |
| Pre-1st CTA recovery: partially recovered | 0 |
| Pre-1st CTA recovery: unrecovered | 100 |


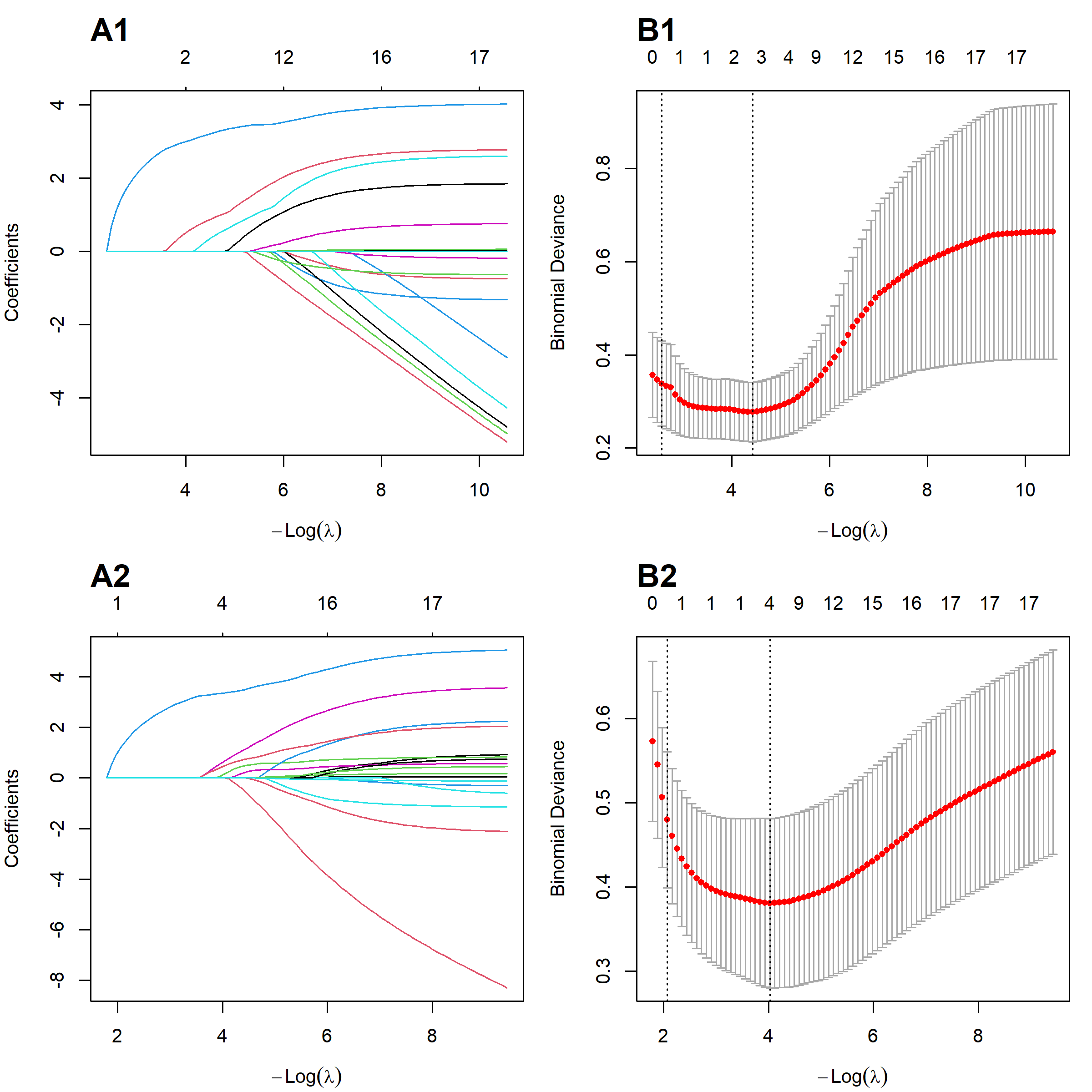


**Figure S1.** LASSO regression for predictor selection in PC-AKI models. A1–B1, results based on temporally split training set: (A1) LASSO coefficient paths; (B1) cross-validated LASSO selection. A2–B2, results based on randomly split training set: (A2) LASSO coefficient paths; (B2) cross-validated LASSO selection.


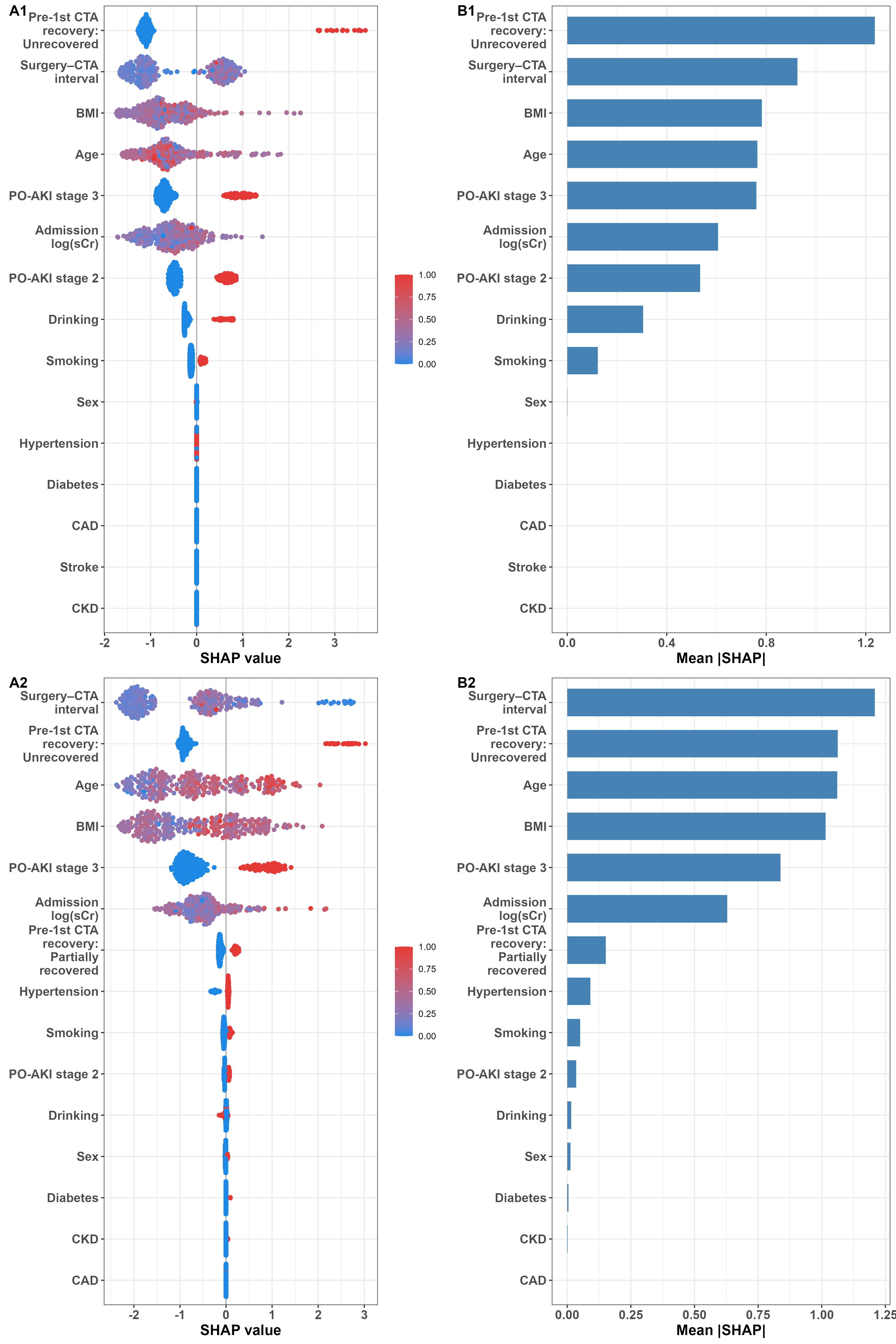


**Figure S2.** XGBoost feature selection for PC-AKI prediction using SHAP values. A1–B1, temporally split training set: (A1) SHAP summary (beeswarm) plot; (B1) SHAP feature importance plot. A2–B2, randomly split training set: (A2) SHAP summary plot; (B2) SHAP feature importance plot. Predictors with cumulative SHAP contribution ≥80% were selected.
